# Modulation of Canine Adipose-Derived Mesenchymal Stem/Medicinal Signalling Cells with Ascorbic Acid: Effect on Proliferation and Chondrogenic Differentiation on Standard Plastic and Silk Fibroin Surfaces

**DOI:** 10.3390/bioengineering11050513

**Published:** 2024-05-20

**Authors:** Metka Voga

**Affiliations:** Veterinary Faculty, University of Ljubljana, Gerbičeva 60, 1000 Ljubljana, Slovenia; metka.voga@vf.uni-lj.si

**Keywords:** mesenchymal stem cells, proliferation, differentiation, ascorbic acid, silk fibroin, dog

## Abstract

Ascorbic acid (AA) plays a crucial role in both the proliferation and chondrogenic differentiation potential of mesenchymal stem/medicinal signalling cells (MSCs); these are both key aspects of their general therapeutic use and their increasing use in veterinary medicine. Current immunomodulatory therapies require efficient expansion of MSCs in the laboratory, while emerging tissue regeneration strategies, such as cartilage or bone repair, aim to use differentiated MSCs and modulate the expression of chondrogenic and hypertrophic markers. Our aim was to investigate whether the addition of AA to the growth medium enhances the proliferation of canine adipose-derived MSCs (cAMSCs) grown on standard plastic surfaces and whether it affects chondrogenic differentiation potential on silk fibroin (SF) films. We assessed cell viability with trypan blue and proliferation potential by calculating population doubling. Chondrogenic induction on SF films was assessed by Alcian blue staining and gene expression analysis of chondrogenic and hypertrophic genes. The results showed that growth medium with AA significantly enhanced the proliferation of cAMSCs without affecting cell viability and modulated the expression of chondrogenic and hypertrophic genes of cAMSCs grown on SF films. Our results suggest that AA may be used in growth medium for expansion of cAMSCs and, at the same time, provide the basis for future studies to investigate the role of AA and SF in chondrogenic differentiation of MSCs.

## 1. Introduction

Mesenchymal stem/medicinal signalling cells (MSCs) are undifferentiated cells with self-renewal and multilineage differentiation capabilities that have in recent years been recognised as promising therapeutic agents for some hard-to-treat diseases. MSCs are increasingly used in not only human but also veterinary medicine. In dogs, cats, and horses, the most common patients in veterinary medicine, stem cells are mainly used to treat orthopaedic diseases such as osteoarthritis. However, the focus of treatment is increasingly expanding to other areas such as diseases of the dental and digestive tract, liver, kidneys, heart, respiratory tract, neuromuscular system, skin, and reproductive system [1]. The therapeutic potential of MSCs is ascribed to their secretion of immunomodulatory and trophic bioactive factors [2]. To provide sufficient numbers of undifferentiated MSCs for treatment, MSCs must be multiplied in the laboratory. The proliferative capacity of MSCs is therefore a crucial aspect of their therapeutic potential. Since MSCs rarely or never differentiate into tissue cells in vivo [3,4], in vitro differentiated MSCs are also being exploited as an alternative cell source in tissue regeneration. In orthopaedics, MSCs differentiated into chondrocytes could potentially replace chondrocytes in autologous chondrocyte implantation or matrix-induced autologous chondrocyte implantation [5], whereas hypertrophic MSCs could contribute to bone formation, as chondrocyte hypertrophy is an intermediate stage in the process of chondrocyte differentiation into osteogenic cells during endochondral ossification [6,7,8]. Modulation of proliferation and chondrogenic differentiation of canine MSCs could therefore help navigate the future preparation of MSCs for therapeutic use in veterinary medicine.

Both proliferation and differentiation potential can be influenced by ascorbic acid (AA). AA is a water-soluble vitamin that is synthesised in the body of most animals (including dogs). It is presumed that species capable of synthesising AA themselves may also respond in vitro to AA [9]. The composition of the cell culture medium could therefore play an important role in determining the potency of MSCs. Although the composition of the media generally differs from laboratory to laboratory and varies depending on the application or research aims, the cell culture medium usually consists of nutrient-providing media such as Dulbecco’s modified Eagle’s medium (DMEM) supplemented with 10% foetal bovine serum (FBS) and antibiotics [10]. For the cultivation of human MSCs, cell culture media are usually formulated without AA [10,11], whereas the addition of AA for the cultivation of animal MSCs (e.g., canine or feline MSCs) is variable. The effect of AA on MSCs is poorly understood, albeit several studies have shown that AA affects cell proliferation [10,12,13,14]. AA is used as one of the components in chondrogenic and osteogenic differentiation media [15,16,17] and has been shown to affect both chondrogenic and osteogenic differentiation of MSCs from different species [17,18].

The aim of our study was to investigate canine adipose-derived MSCs (cAMSCs) under the influence of AA. We investigated whether AA improves the proliferation of cAMSCs grown on standard plastic surfaces and affects differentiation toward the chondrogenic lineage on silk fibroin (SF) films. SF is one of the most promising biomaterials for tissue engineering due to its biocompatibility, excellent mechanical properties [19], and ability to assume various forms such as films, hydrogels, tubes, sponges, fibres, and composites [20]. We have shown in our previous study [21] that SF can be used to induce the differentiation of cAMSCs towards chondrogenesis when cultured in a standard growth (and not differentiation) medium. Since some other biomaterials such as PEG–chitosan composite [22], hyaluronic acid [23], and gelatine–hydroxyapatite composite [24] have been shown to regulate the hypertrophy of MSCs, in the present study, we chose to differentiate MSCs on SF films as this is not a standard procedure and could also potentially lead to different gene expression results than chondrogenesis induced in differentiation medium on a plastic surface. In the present study, we have shown that AA significantly affects the proliferation potential of cAMSCs on plastic surfaces and also modulates the expression profile of chondrogenic and hypertrophic genes during chondrogenic differentiation induced by silk fibroin.

## 2. Materials and Methods

### 2.1. Animals and Adipose Tissue Collection

Adipose tissue was collected by veterinarians in veterinary clinics. All dogs were privately owned patients and were under general anaesthesia for adipose tissue sampling for stem cell therapy of OA, provided by Animacel Ltd. Subcutaneous adipose tissue (<1 cm^3^) was collected aseptically from the dorsal region between the shoulder blades of each dog. Samples were collected from ten different dogs aged 6–14 years (mean age 9.4 years) of different breeds and both sexes. The owners of all dogs gave their written consent for the use of the collected adipose tissue and cells for research purposes. All experiments were performed in accordance with Slovenian legislation and the official opinion of the Administration of the Republic of Slovenia for Food Safety, Veterinary Sector, and Plant Protection, which is responsible for granting ethical approvals for animal experiments, and no further approval of the Ethics Committee was required in accordance with Slovenian legislation.

### 2.2. Isolation of cAMSCs

The adipose tissue was washed with Dulbecco’s phosphate-buffered saline (DPBS, Gibco, Grand Island, NY, USA) and cut into small pieces with a scalpel. The adipose tissue was then incubated overnight at 37 °C in Dulbecco’s modified Eagle’s medium (DMEM, Gibco, USA) containing 0.1% collagenase type II (Sigma–Aldrich, Taufkirchen, Germany). The digested tissue was centrifuged at 1600 rpm for 4 min, and the supernatant was discarded. The cell pellet was resuspended in a proprietary cell culture medium containing DMEM and 10% foetal bovine serum (FBS, Gibco, USA). The cell suspension was plated into 6-well plates (TPP, Trasadingen, Switzerland) and cultured at 37 °C in a 5% CO_2_ incubator. The cell culture medium was changed every 2–3 days. After reaching 70–90% confluence, cells were trypsinized and multiplied by seeding 10^4^ cells per cm^2^ into a larger (T75) cell culture flask. After a sufficient number of cells were obtained, the cells were further processed for expression of surface markers, potential for differentiation into multiple cell lineages, and culturing of experimental cell cultures.

### 2.3. Flow Cytometry for Cell Surface Markers

Flow cytometry was performed on the cells to evaluate the expression of cell surface markers. Antibodies against the MSC markers CD44, CD90, CD29, and CD34. A total of 1 × 10^6^ cells from passage 3 was used. Following trypsinization, cells were counted, centrifuged (240× *g* for 4 min), and washed twice with DPBS. Cells were stained with the following antibodies for canine ADMSCs: allophycocyanin (APC) conjugated against CD44 (antibody clone IM7, 103012, Biolegend, San Diego, CA, USA), phycoerythrin (PE) conjugated against CD90 (antibody clone YKIX337.217, 12-5900-42, eBioscience, San Diego, CA, USA), and fluorescein isothiocyanate (FITC) conjugated against CD29 (antibody clone MEM-101A, MA1-19566, ThermoFisher Scientific, Waltham, MA, USA) and CD34 (antibody clone 581, 60013FI, Stemcell Technologies, Cambridge, MA, USA). For antibody titration, 1, 2, 3, 4, 5, and 10 μL of each antiserum per 100 μL of 1 × 10^6^ cells was used. Appropriate dilutions of antibodies used for staining are shown in Table 1. Cells were then vortexed, incubated at room temperature in the dark for 10 min, washed twice with DPBS, vortexed, and centrifuged again (500× *g* for 5 min). The supernatant was decanted. Finally, cells were resuspended in 100 μL of DPBS for FACS analysis. The exclusion of non-viable cells was performed by staining cells with propidium iodide solution (Molecular Probes, Eugene, OR, USA). Experimental settings were set up using unstained cells and single-colour stain. A minimum of 20,000 events was recorded. Cells were analysed with a BD FACSAria III flow cytometer (BD Bioscience, Franklin Lakes, NJ, USA). FACSDiva 9.4 software (BD Bioscience) was used for FACS data analysis.

### 2.4. Trilineage Differentiation Potential

Differentiation potential was assessed by inducing cell differentiation into adipocytes, osteocytes, and chondrocytes. For adipogenic differentiation, 4 × 10^4^ cells were seeded in 12-well plates. The day after seeding, the cell culture medium was removed. The adipogenic medium (StemPro Adipogenesis Differentiation Kit, Gibco, USA) was added and changed every 2–3 days. The cell culture medium was added to the wells, which served as negative controls. Adipogenic differentiation was analysed after 14 days of cultivation using Oil-red-O staining (Sigma–Aldrich) according to the standard procedure. For osteogenic differentiation, 4 × 10^4^ cells were seeded in 12-well plates. After 90–100% confluence was reached, the cell culture medium was removed. Osteogenic medium (StemPro Osteogenesis Differentiation Kit, Gibco, USA) was added and changed every 2–3 days. Osteogenic differentiation was analysed after 14 days of cultivation using Alizarin Red S staining (Sigma–Aldrich) according to the standard procedure. For chondrogenic differentiation, micromass cultures were generated by seeding 5 μL droplets containing 4 × 10^4^ cells into the middle wells of a 12-well plate. After the micromass cultures were cultured for 6 h under high humidity, a chondrogenic medium (StemPro Chondrogenesis Differentiation Kit, Gibco, USA) was added to the culture vessels. The cell culture medium was added to the wells, which served as negative controls. The micromass cultures were incubated at 37 °C in an incubator with 5% CO_2_ and a humid atmosphere. The medium was changed every 2–3 days. Chondrogenic differentiation was analysed after 14 days of cultivation using Alcian blue staining (Sigma–Aldrich) according to a standard procedure. The differentiated cells were visualized under a light microscope.

### 2.5. Cell Viability and Proliferation Potential Assay

The proliferation potential assay was performed on cells cultured in medium with or without AA (0.2 mM) in the form of L-ascorbic acid-2-phosphate (AA; Sigma–Aldrich). We decided on a concentration of 0.2 mM AA based on some studies with human MSCs [11,12,13] and preliminary unpublished data from previous internal studies kindly provided by Animacel Ltd (Ljubljana, Slovenia). Proliferation potential was assessed from the fourth to the seventh passage. Every 72 h, cells were trypsinized and seeded at a density of 1 × 10^4^ cells per cm^2^ as a new passage. At each passage, from the fourth to the seventh, the number of cells at seeding and harvest was determined with a haemocytometer, and cell viability was assessed with trypan blue dye. PD and CPD were calculated using the following formulas:

$$\mathrm{PD}=\log\left(\frac{\mathrm{Nt}}{N0}\right)3.32$$

$$\mathrm{CPD}=\mathrm{PD}\left(P4\right)+\mathrm{PD}\left(P5\right)+\mathrm{PD}\left(P\&\right)+\mathrm{PD}\left(P7\right)$$

PD is the number of doublings of a population at one passage, Nt is the number of cells at harvesting, and N0 is the number of cells at seeding [25]. CPD is the cumulative PD of all passages. All experiments were repeated 5 times.

### 2.6. Cell Culturing on SF Films

For all experiments with SF, commercial 50 mg/mL SF solution (Advanced Biomatrix, Carlsbad, CA, USA) was used. For SF films, SF at a concentration of 12.5 mg/mL was prepared by diluting the SF with mqH_2_O, pouring 600 μL of SF solution (12.5 mg/mL) into the wells of 6-well and 12-well cell culture plates, and then air drying overnight. The films were then incubated in 70% ethanol for 10 min. In the final step, the films were washed thoroughly with DPBS. After sufficient numbers of cells were obtained at passage 3, cells were trypsinized and seeded in the 4th passage in 6-well and 12-well cell culture plates coated with SF films at a seeding density of 1 × 10^4^ cells per cm^2^. Based on our previous study, conducted on SF films (in which we showed that differentiation of cAMSCs towards a chondrogenic lineage can occur on SF films when cultured in a cell growth medium), we cultured the cells for 3 and 7 days in a cell growth medium with or without AA. On days 3 and 7, cells from the 12-well cell culture plates were stained with Alcian blue, while cells from the 6-well cell culture plates were used for isolation of RNA. Cell cultures were named accordingly (Table 2).

### 2.7. Alcian Blue Staining and Analysis

Alcian blue staining was used for cells cultured in the 12-well cell culture plates coated with SF films. At 3 and 7 days after culturing, the medium was removed from the culture vessels. Wells were rinsed twice with DPBS, and cells were fixed with 4% paraformaldehyde solution for 1 h at room temperature. Following fixation, wells were rinsed twice with dH_2_O and incubated overnight in 1% Alcian blue stain, prepared in 0.1 N HCl. The next day, wells were rinsed three times with 0.1 N HCl followed by DPBS to neutralize the acidity. Wells were examined under the inverted light microscope. All experiments were repeated 6 times.

### 2.8. Light Microscopy and ImageJ Analysis

An inverted light microscope (Nikon Eclipse TS100, Nikon, Tokyo, Japan) with a Nikon Digital Sight DS-U2 camera was used for Alcian staining analysis. Images were acquired using the NIS-Elements D3.2 Live quality program. Images of the cells were taken at 4000× magnification. Seven fields of view in a well of a 12-well plate were randomly selected and quantitatively analysed in the ImageJ program [26]: the background was subtracted, and colours were separated using the Color Deconvolution2 plugin [27,28], selecting the Alcian blue and H vector and 8-bit transmission. The Alcian blue image was then further segmented using the DynamicThreshold_1d plugin [29], displaying (max + min)/2 images. The total area of particles larger than 10 μm^2^ and the average particle size were measured.

### 2.9. RNA Isolation

RNA was isolated from SF cells, positive control cells, and negative control cells. Cells were detached from the surfaces with a cell scraper. The cell suspension was removed from the wells and centrifuged at 1600 rpm for 4 min. The cell pellet was resuspended in 300 μL TRIzol (ThermoFisher) and frozen at −80 °C. The cell pellet was homogenized in 300 μL TRIzol (ThermoFisher) using a homogenizer (IKA T10 basic, Staufen, Germany), and 700 μL TRIzol was added to a total volume of 1 mL TRIzol. Extraction of total RNA was performed with TRIzol according to the manufacturer’s protocol. The amount of total RNA extracted was measured using a UV spectrophotometer (ThermoFisher) at a wavelength of 260/280 nm.

### 2.10. Reverse Transcription Quantitative Polymerase Chain Reaction

Two-step reverse transcription quantitative polymerase chain reaction (RT qPCR) was performed for SF cells, negative control cells, and positive control cells. First, 1 μg total RNA of each sample was transcribed into cDNA using the High Capacity cDNA Reverse Transcription Kit with RNase Inhibitor (ThermoFisher) according to the manufacturer’s protocol. Negative reverse transcription controls were included in each PCR run. All reactions were performed in a total volume of 20 μL. Reverse transcription conditions were as specified in the manufacturer’s protocol: 25 °C for 10 min, 37 °C for 120 min, and 85 °C for 5 min. In the second step, relative quantification was performed using TaqMan Universal PCR Master Mix with UNG (ThermoFisher) and TaqMan gene expression assays, SOX9, ACAN, COMP, Col2A1, Col1A1, MMP13, RUNX2, and MEF2C. Because Col10 was not commercially available for dog species, RUNX2 was used instead, as it is a direct transcriptional factor of COL10A1. COL1A1 was included among the hypertrophic genes because it is not only a main component of the fibrocartilage characteristic of pathological situations in joints such as those with OA but also a characteristic of endochondral ossification and new bone formation. TBP was used as a reference gene (Table 3; ThermoFisher). All qPCR amplifications were performed in triplicate in a total volume of 20 μL. A total of 20 ng of cDNA was used as a template. Amplification was performed in 96-well plates with a Light Cycler 96 (Roche Life Science, Raleigh, NC, USA) using the following program: 50 °C for 2 min, 95 °C for 10 min, and 40 cycles at 95 °C for 15 s, 60 °C for 60 s. All experiments were repeated 6 times for positive control cells and 10 times for SF cells and negative control cells.

### 2.11. Statistical Analysis

All statistical analyses were performed with GraphPad Prism version 9.5.0 for Windows, GraphPad Software, San Diego, CA, USA, www.graphpad.com (accessed on 15 April 2024).

The viability and proliferation potential experiments were repeated five times with cells from five different dogs. Normality and lognormality were checked with the Kolmogorov–Smirnov test. Viability and proliferation potential were analysed with repeated measures two-way ANOVA using medium and passages as within factors. To clarify differences between pairs, Šidák’s multiple comparison test was performed.

The gene expression analysis experiment was repeated ten times with cells from different dogs for the SF groups and the negative control and six times with cells from different dogs for the positive control. All RT qPCR experiments were performed in triplicate. The efficiency-corrected double-delta Ct method was used to normalise gene expression values [42]. The normality and lognormality were checked with the Kolmogorov–Smirnov test. The data for gene expression analysis for all genes were log-transformed before statistical analysis. First, the expression of each gene in all groups of SF cells and the positive control was compared with the expression in the negative control. The expression of SOX9, ACAN, COL2, COL1, MMP13, RUNX2, and MEF2C was analysed using one-way ANOVA, and the expression of COMP was analysed using the Kruskal–Wallis nonparametric test. Post hoc Tukey’s multiple-comparisons and Dunn’s multiple-comparisons tests were used, respectively, to clarify differences between specific pairs. Second, a separate statistical analysis was performed for only four groups of SF cells to examine the effects of the type of medium and duration of cultivation specifically on these cell groups. To analyse the expression of SOX9, ACAN, COL2, COL1, MMP13, RUNX2, and MEF2C between the SF groups, two-way ANOVA was used, and to analyse the expression of COMP, the Friedman test was performed. Post hoc Šidák’s and Dunn’s multiple comparison tests were performed, respectively, to clarify the differences between the pairs.

In the experiments with Alcian blue staining, the effects of the type of medium and the duration of cultivation were investigated for all four SF groups. The experiment was repeated six times with cells from different dogs. Normality and lognormality were checked with the Kolmogorov–Smirnov test. Data were log-transformed before Alcian blue staining analysis. A two-way repeated-measures comparison was performed (ANOVA) using the medium and time of culture as within factors. Šidák’s multiple comparison test was performed to further clarify differences.

Statistical significance was determined with *p* < 0.05.

## 3. Results

### 3.1. Isolation and Characterization of cAMSCs

**Figure 1:**
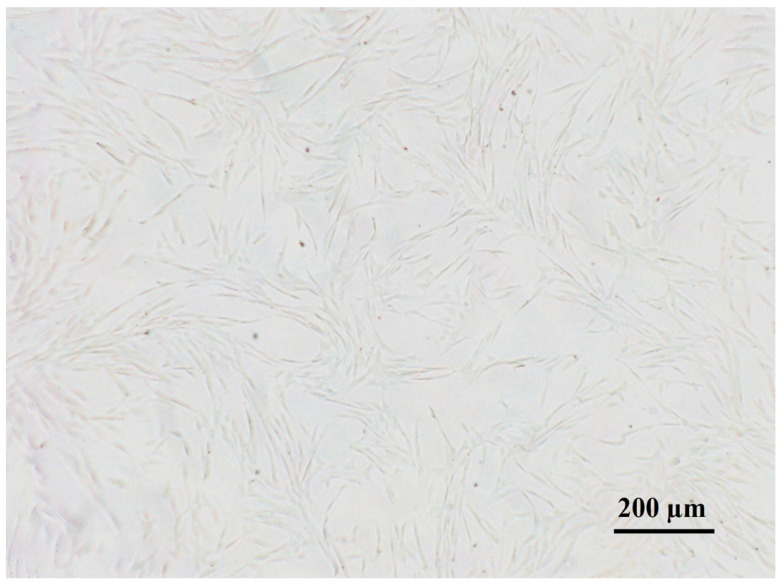
Morphology of cells grown on a standard plastic surface. cAMSCs from passage 3 are spindle-shaped with typical fibroblast-like morphology.

Adipose tissue was successfully collected from all animals. Under a light microscope, the cells from passage 3 appeared spindle-shaped with a fibroblast-like morphology (Figure 1).

### 3.2. Flow Cytometry for Surface Marker Expression

**Figure 2:**
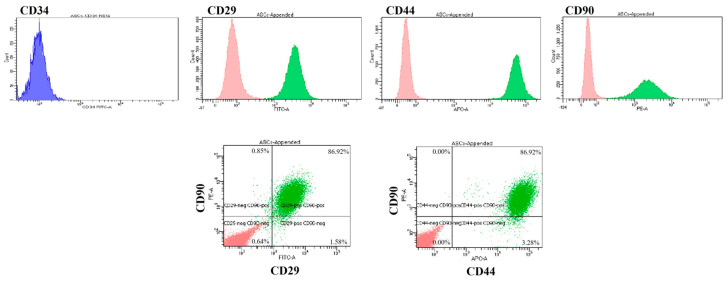
Expression of cAMSC surface markers. In the upper row are histograms of the negative marker CD34-FITC (blue) and the positive markers CD29-FITC, CD44-APC, and CD90-PE. In the lower row, dot plots of the positive stem cell markers CD29, CD44, and CD90 are presented. Blue and green are cells labelled with antibodies. Images of negative control cells that were not labelled with antibodies (red) are appended to the images of the labelled (green) cells in both histograms and dot plots. In the dot plots, the percentage of negative control cells is 100% (not shown), while the percentage of labelled cells is shown in each quadrant of the dot plots.

Flow cytometry was performed at passage 3 with conjugated primary antibodies against the positive surface markers CD44-APC, CD90-PE, and CD29-FITC and the negative surface marker CD34-FITC. The cells were positive for CD44, CD90, and CD29 but negative for CD34 (Figure 2).

### 3.3. Multilineage Differentiation Potential

**Figure 3:**
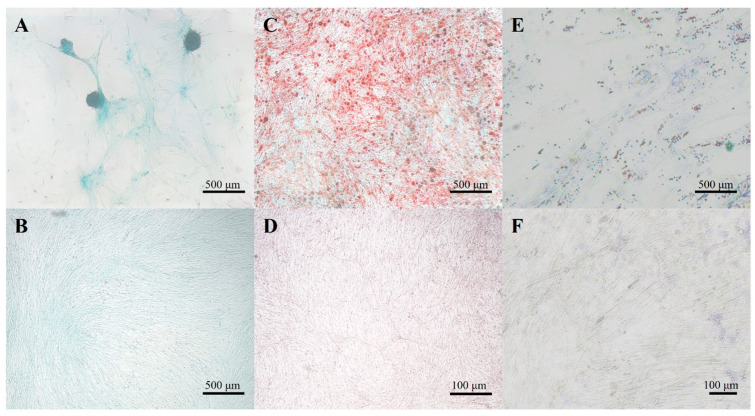
Trilineage differentiation potential of cAMSCs. cAMSCs successfully differentiated into chondrogenic, osteogenic, and adipogenic lineages. Chondrogenesis is indicated by the formation of chondrogenic nodules stained blue with Alcian blue (**A**). Mineral deposits in the extracellular matrix stained red with alizarin red S indicate osteogenesis (**C**). In adipogenic differentiation, intracellular lipid droplets are stained red with Oil-Red-O (**E**). Corresponding negative controls are shown below (**B**,**D**,**F**).

cAMSCs were able to differentiate into adipocytes, osteocytes, and chondrocytes when cultured in specific differentiation media. After adipogenic differentiation, intracellular lipid droplets stained red with Oil-red-O. After osteogenic differentiation, mineral deposits in the extracellular matrix stained red with Alizarin-Red-S, and after chondrogenic differentiation, proteoglycans in the extracellular matrix of layered cell clusters stained positive with Alcian blue (Figure 3).

### 3.4. Cell Viability and Proliferation Potential

**Figure 4:**
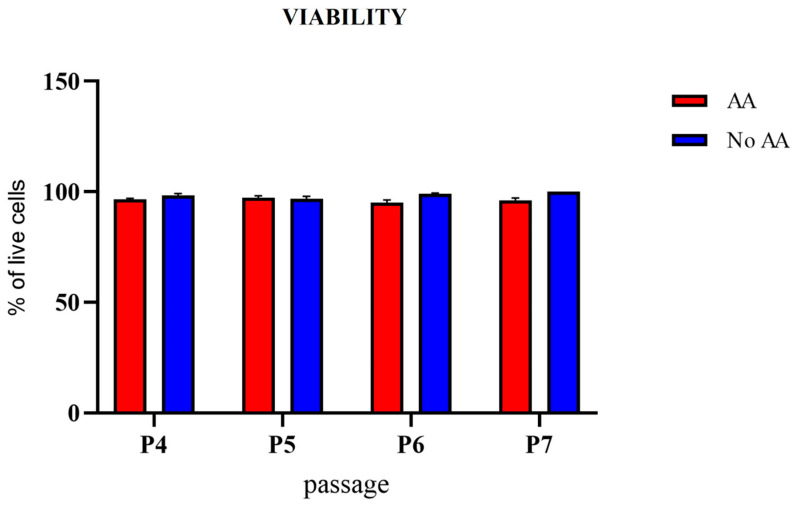
Viability of cAMSCs grown on a standard plastic surface in culture medium with or without AA. Cell viability was similar in both groups of cells. Results are presented as the mean ± SEM.

**Figure 5:**
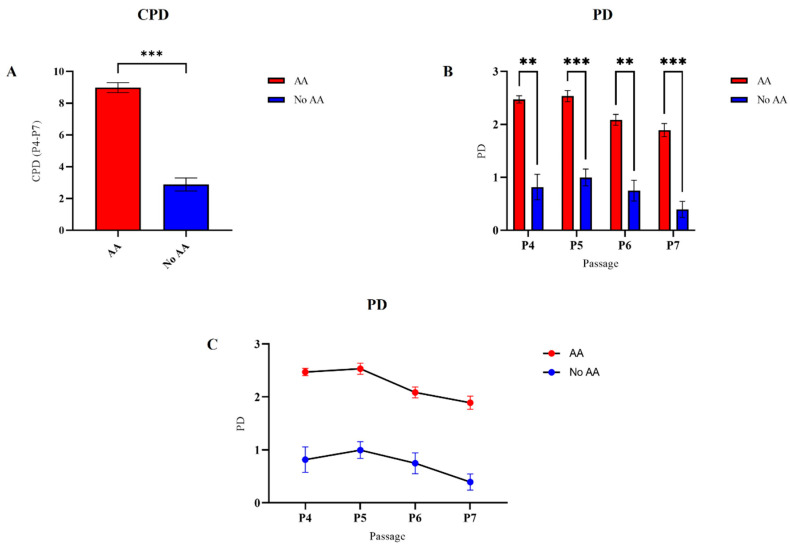
The proliferation potential of cAMSCs grown on a standard plastic surface in culture medium with or without AA. CPD was statistically significantly higher in cAMSCs grown in medium with AA than in medium without AA ((**A**), *** *p* < 0.001). The difference in PD between groups was present in all passages and was higher in P5 and P7 ((**B**), *** *p* < 0.001) than in P4 and P6 ((**B**), ** *p* < 0.01). The pattern of PD between passages was similar in both cell groups (**C**).

The viability and proliferation potential of cAMSCs cultured in medium with or without AA were measured from the fourth to the seventh passage. Viability was similar for SF-AA and SF-NoAA in all passages (Figure 4). CPD was statistically significantly higher in cAMSCs cultured in medium with AA than in medium without AA (*p* < 0.001; Figure 5A). PD of cells grown in medium with or without AA was significantly different in each passage (*p* < 0.01 for P5 and P7, *p* < 0.001 for P4 and P6; Figure 5B). The curve of PD between passages was similar for cells grown in medium with and without AA (Figure 5C).

### 3.5. Alcian Blue Staining

**Figure 6:**
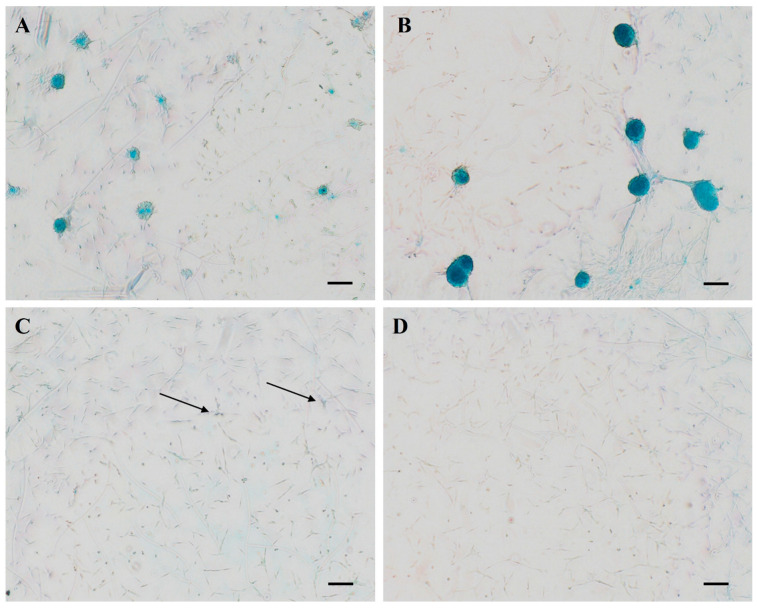
Morphology and Alcian blue staining of cAMSCs grown on SF films. In SF-AA-3D cAMSCs (**A**), small clusters of cells resembling a nodule have formed and stained blue with Alcian blue. In SF-AA-in 7D cAMSCs (**B**), the nodules appear larger and more intensely stained. In SF-NOAA-3D (**C**) and SF-NOAA-7D cAMSCs (**D**), there is a tendency to form cell clusters in which positive Alcian blue staining is observed in some parts (arrows). However, larger blue nodules, as seen for SF-AA, did not form at any time point. Scale bars are 100 μm.

**Figure 7:**
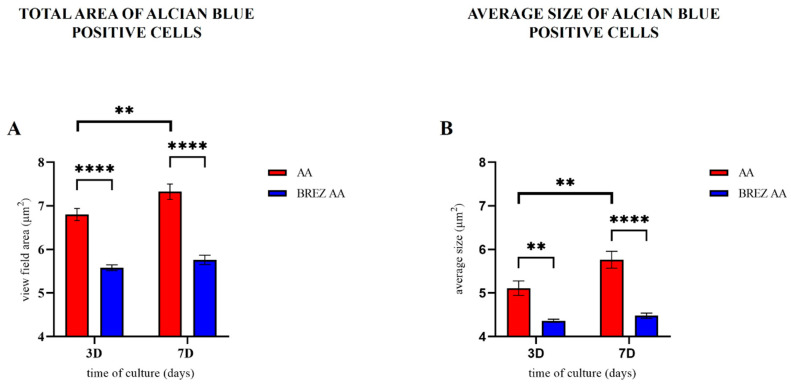
Total area and average size of Alcian blue-positive cAMSCs grown on SF films in culture medium with or without AA. The total area of cells positively stained with Alcian blue was statistically significantly higher in cells grown in medium containing AA than in cells grown in medium without AA after both 3 and 7 days of cultivation ((**A**), **** *p* < 0.0001). Similarly, the average size of Alcian blue-positive particles was larger in cells grown in medium containing AA than in cells grown in medium without AA, both after 3 (** *p* < 0.01) and after 7 days (**** *p* < 0.0001) of cultivation (**B**). For cells grown in medium containing AA, the total area of Alcian blue staining and the size of blue particles increased from 3 to 7 days of cultivation (** *p* < 0.01), whereas for cells grown in medium without AA, the total area and the average size of particles were similar for 3 and 7 days (**A**,**B**).

In SF-AA-3D cAMSCs (Figure 6A), small clusters of cells resembling a nodule formed and stained blue with Alcian blue. In SF-AA-7D cAMSCs (Figure 6B), the nodules were larger and more intensely stained. In SF-NoAA-3D (Figure 6C) and SF-NoAA-7D cAMSCs (Figure 6D), there was a tendency for cell clusters to form, but larger nodules did not form. The total area of cells positively stained with Alcian blue was statistically significantly higher in cells grown in medium containing AA than in cells grown in medium without AA after both 3 and 7 days of cultivation (*p* < 0.0001; Figure 7A). Similarly, the average size of Alcian blue-positive particles was larger in cells grown in medium containing AA than in cells grown in medium without AA, both after 3 (*p* < 0.01) and after 7 days (*p* < 0.0001) (Figure 7B). For cells grown in medium containing AA, the total area of Alcian blue staining and the size of blue particles increased from 3 to 7 days of cultivation (*p* < 0.01; Figure 7A,B), while for cells grown in medium without AA, the total area and average size of particles were similar at both time points.

### 3.6. Gene Expression of Chondrogenic and Hypertrophic Marker Genes

Chondrogenic (COL2, SOX9, ACAN, and COMP) and hypertrophic (COL1, MMP13, RUNX2, and MEF2C) genes were analysed.

First, expression of chondrogenic and hypertrophic genes in all SF groups (SF-AA-3D, SF-NoAA-3D, SF-AA-7D, and SF-NoAA-7D) and the positive control was compared with expression in the negative control. Second, analysis of four groups of SF cells only was conducted to examine the effects of AA and duration of cultivation on these groups of cells specifically.

**Figure 8:**
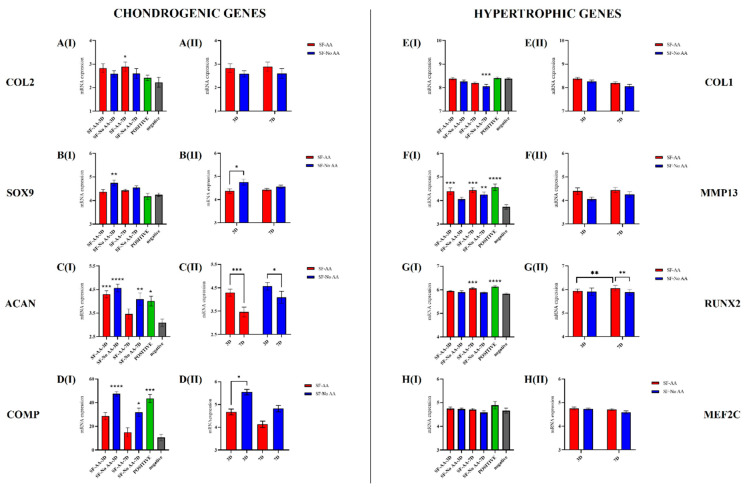
Expression of chondrogenic and hypertrophic genes in cAMSCs grown on SF films in culture medium with or without AA. In each of the two groups of genes (chondrogenic and hypertrophic genes), the plots on the left (**I**) represent the comparison of SF-AA-3D, SF-NoAA-3D, SF-AA-7D, SF-NoAA-7D, and the positive control with the negative control. The graphs on the right (**II**) represent comparisons between the four groups of SF cells cultured for 3 and 7 days in medium with or without AA (SF-AA-3D, SF-NoAA-3D, SF-AA-7D, and SF-NoAA-7D). *p*-values are as follows: *: *p* < 0.05, **: *p* < 0.01, ***: *p* < 0.001, and ****: *p* < 0.0001.

Chondrogenic genes: COL2: Compared with the negative control, the expression of COL2 was significantly higher in SF-AA-7D (*p* < 0.05; Figure 8(AI)). When comparing only SF cells, there were no differences between SF cells with respect to the different media or times of cultivation (Figure 8(AII)). SOX9: Compared with the negative control, the expression of SOX9 was significantly higher in SF-NoAA-3D (*p* = 0.001; Figure 8(BI)). When comparing only SF cells, the expression of SOX9 on day 3 of cell culture was significantly higher in SF-NoAA than in SF-AA (*p* < 0.05; Figure 8(BII)). ACAN: Compared to the negative control, the expression of ACAN was higher in SF-AA-3D (*p* < 0.001), SF-NoAA-3D (*p* < 0.0001), SF-NoAA-7D (*p* < 0.01), and the positive control (*p* < 0.05) but similar in SF-AA-7D (Figure 8(CI)). When comparing only SF cells, expression of ACAN was higher on day 3 than on day 7 of cell culture in both SF-AA and SF-NoAA (*p* < 0.001 and *p* < 0.05, respectively; Figure 8(CII)). COMP: Compared to the negative control, the expression of COMP was significantly higher in SF-NoAA-3D (*p* < 0.0001), SF-NoAA-7D (*p* < 0.05), and the positive control (*p* < 0.001) (Figure 8(DI)). When comparing only SF cells, the expression of COMP was significantly higher in SF-NoAA than in SF-AA on day 3 of cell culture (*p* < 0.05; Figure 8(DII)).
Hypertrophic genes: COL1: Compared with the negative control, COL1 expression was significantly lower in SF-NoAA-7D (*p* < 0.001; Figure 8(EI)). There were no differences between SF cells with respect to the different media or times of culturing when comparing SF cells only (Figure 8(EII)). MMP13: Expression of MMP13 in SF-NoAA-3D was similar to that in the negative control. In all other cell groups, expression was higher and significantly different from the negative control (*p* < 0.001 for SF-AA-3D, *p* = 0.0001 for SF-AA-7D, *p* < 0.01 for SF-NoAA-7D, and *p* < 0.0001 for the positive control; Figure 8(FI)). There were no differences between SF cells with respect to the different media or times of culturing when comparing SF cells only (Figure 8(FII)). RUNX2: Compared with the negative control, the expression of RUNX2 was significantly higher in SF-AA-7D (*p* < 0.001) and the positive control (*p* < 0.0001), while the expression of RUNX2 in the other cell groups was similar to that in the negative control (Figure 8(GI)). When comparing only SF cells, the expression of RUNX2 was significantly higher in SF-AA on day 7 than on day 3 (*p* < 0.01), and on day 7, the expression was higher in SF-AA than in SF-NoAA (*p* < 0.01) (Figure 8(GII)). MEF2C: There were no differences in MEF2C expression between cell groups (Figure 8H).

## 4. Discussion

The proliferation and differentiation of MSCs are a key aspect of their therapeutic potential, either for existing therapies with undifferentiated cells or for emerging therapies with differentiated MSCs for cartilage or bone regeneration. In the present study, we investigated the effect of AA on the proliferation and differentiation of cAMSCs. We have shown that AA in a cell culture medium significantly improves the proliferation of cAMSCs on a standard plastic surface without affecting their viability and modulates the expression of chondrogenic and hypertrophic genes during chondrogenic differentiation when grown on SF.

For characterization of cAMSCs, we have differentiated cells into adipocytes, chondrocytes, and osteocytes and investigated the expression of MSC surface markers and shown that cAMSCs express the positive MSC markers CD29, CD90, and CD44 but do not express the negative MSC marker CD34. For defining human MSCs, minimal criteria have been set by the International Society for Cellular Therapy, stating that MSCs must be able to differentiate into osteoblasts, adipocytes, and chondroblasts. In addition, MSCs must express CD105, CD73, and CD90 and lack the expression of CD45, CD34, CD14 or CD11b, CD79a or CD19, and HLA class II (32) surface markers. However, there are no minimum criteria set to define animal MSCs based on surface antigens as there are for human MSCs. We therefore chose as positive markers the antibodies against CD29, CD44, and CD90, which have been shown to be consistently expressed on canine MSCs [43,44,45].

For the cultivation of human MSCs, cell culture media are usually formulated without AA [10,11], whereas the addition of AA for the cultivation of animal MSCs (e.g., canine or feline MSCs) varies. The addition of AA to cell culture media has been shown to increase the proliferation capacity of bovine foetal chondrocytes [46] and human MSCs [10,12,13,14] when grown on standard tissue culture polystyrene or decellularized gelatine-based extracellular matrix (dECM) [47]. The beneficial effect of AA on the proliferation potential of MSCs has been attributed to increased activity of hypoxia-inducible factor 1-α (HIF1-α) hydrolase, which represses HIF1-α transcription and associated mitochondrial activation [10]. Compared to human MSC studies, very few studies have investigated the effect of AA on canine MSC proliferation [48]. The results of our study are consistent with the results of studies performed on human MSCs, as we have shown that AA promotes the proliferation of cAMSCs. Although the recommended concentration of AA is 0.05 to 0.2 mM [11], data from the literature vary on the dose-dependent effect of AA on cell proliferation. While some studies have reported no differences in cell proliferation depending on different doses of AA [10,47], others [12,14] have shown that higher doses are negatively correlated with cell yield due to oxidative toxicity. However, it has been suggested that oxidative cytotoxicity is enhanced by lower cell seeding density and therefore could be reversed or prevented by seeding cells at higher density or by co-administration of another antioxidant [14,49]. In our study, we used 0.2 mM AA in medium containing AA and seeded the cells at a density of 1 × 10^4^/cm^2^. Since the viability of cells in medium with or without AA was similar for all passages, the dose of AA used did not appear to have a cytotoxic effect on cAMSCs at the given cell density. Summarizing the cell viability and proliferation potential of cAMSCs, AA appears to promote the proliferation of cells cultured in AA-enriched medium without compromising cell viability and may therefore be used in a growth medium when faster expansion of cAMSCs is desired.

AA plays an important role in cell differentiation. It is an essential cofactor for the enzymes lysyl hydroxylase and prolyl hydroxylase and essential in collagen biosynthesis [50]. In the absence of AA, collagen cannot form into a proper helical structure because proline is not hydroxylated [51]. AA is therefore used as one of the components in chondrogenic and osteogenic differentiation media [15,16,17]. Some biomaterials have been shown to reduce the hypertrophy of MSCs, e.g., PEG–chitosan composite [22], hyaluronic acid [23], and gelatine–hydroxyapatite composite [24]. The non-standard chondrogenic differentiation process of MSCs using SF may therefore provide a promising opportunity to study the expression of chondrogenic and hypertrophic genes, as it may differ from the expression of genes on standard plastic surfaces.

The correlation between SF and chondrogenesis of MSCs has been confirmed in previous studies. Rosadi et al. found that a combination of SF scaffold and platelet-rich plasma (PRP) effectively induced chondrogenesis of human ADMSCs, as evidenced by upregulation of COL2 and ACAN and downregulation of COL1, compared with the negative control [52]. Barlian et al. found that the combination of silk fibroin and silk spidroin promoted chondrogenesis of Wharton jelly–derived human MSCs more effectively than silk fibroin alone. Chondrogenesis was analysed by GAG content, and it was found that the use of cell culture medium supplemented with PRP resulted in higher GAG accumulation compared to medium supplemented with AA [53]. We have previously shown that SF also induces the differentiation of cAMSCs when grown in a standard cell growth medium [21]. In the present study, we therefore used SF as a biomaterial to trigger chondrogenesis of cAMSCs and investigated whether the Alcian blue staining and gene expression of cAMSCs cultured on SF is modulated by AA.

In the first part of the differentiation study, we analysed Alcian blue staining of cAMSCs grown on SF films (SF-cells) in a growth medium optionally supplemented with AA. We found a clear morphological difference between SF-AA and SF-NoAA cells. Whereas SF-AA cells formed chondrogenic nodules, very few were present in SF-NoAA cells, although the tendency to form cell clusters was present. The total area and size of Alcian blue-positive SF cells differed significantly between SF-AA and SF-NoAA cells. Both the total area and the average size of Alcian blue-positive cells were significantly higher in cells cultured with AA than in cells cultured without AA at both 3 and 7 days. Moreover, in cells cultured in medium containing AA, the total area of Alcian blue staining and the size of blue particles increased from 3 to 7 days of cultivation. The results of Alcian blue study are in line with the abovementioned studies, confirming that SF appears to play a role in chondrogenesis of human and animal AMSCs. Further, increased staining intensity and larger nodule size over time suggest that AA supplementation promotes chondrogenic differentiation and extracellular matrix production of cAMSCs grown on SF.

Interestingly, SF in combination with AA has been associated with a hypertrophic phenotype of associated cells also by Fan et al., demonstrating the beneficial effects of incorporating L-ascorbic acid-2-phosphate into silk fibroin, resulting in significant promotion of collagen type I in L929 mouse fibroblasts [54]. In addition, Gandhimathi et al. demonstrated that SF in combination with AA, polycaprolactone, and dexamethasone promoted osteogenic differentiation of human AMSCs, as confirmed by alkaline phosphatase activity and mineralization [55].

We wanted to know whether the addition of AA to the growth medium also modulates the gene expression of cAMSCs cultured in a growth medium on SF films. In the second part of the differentiation study, we analysed the expression of chondrogenic (COL2, SOX9, ACAN, and COMP) and hypertrophic (COL1, MMP13, RUNX2, and MEF2C) genes in cAMSCs grown on SF (SF cells). Cell growth medium was optionally supplemented with AA. Cells were analysed at 3 and 7 days. First, gene expression of SF cells and the positive control was compared with expression in the negative control. Second, SF cells were compared with each other to examine the effects of AA and duration of cultivation on SF films only.

In chondrogenic gene analysis, we have shown that the expression of COL2 was significantly higher in SF-AA-7D compared with the negative control but not in other cell groups. The results of COL2 expression are supported by other studies of chondrocytes cultured in either monolayer or agarose gel, where AA promoted COL2 expression [56,57,58,59], although not in all studies [60]. However, when we focused only on the SF cells, no statistical differences were found between the groups. Therefore, it is difficult to assume that the expression of COL2A1 is due to the combined effect of SF and the addition of AA to the medium.

SOX9 is a transcription factor required for chondrocyte differentiation and expression of chondrocyte-specific marker genes such as COL2A1, ACAN, and COMP [30,61,62]. In the present study, upregulation of SOX9 was detected only when compared with the negative control. This could also explain the high expression of ACAN and COMP in the SF-NoAA-3D cell group. The results of the analysis of SF cells indicate that SF with the addition of AA to the culture medium suppressed the upregulation of SOX9. Herring et al. have shown that expression of ACAN in chondrocytes peaks in the first 3 days of the study, followed by a decline in the next few days [59,63]. In our study, the results were similar. Based on the analysis of SF cells, the expression of ACAN was higher on day 3 than on day 7 of cell culture, in both SF-AA and SF-NoAA, suggesting that the expression of ACAN is higher at earlier time points and then decreases, regardless of the presence of AA. Similar to ACAN, COMP was also repressed in SF cells when AA was added to the medium, as indicated by the upregulation of COMP in all SF-NoAA cells and the positive control compared with the negative control. When comparing SF cells with each other, the upregulation of COMP was higher in SF-NoAA than in SF-AA on day 3.

In addition to chondrogenic gene expression, we also examined hypertrophic gene expression, as AA has previously been associated with a hypertrophic phenotype [64]. It has been reported to induce the maturation of prehypertrophic sternal chondrocytes of chicken embryos (Leboy et al. 1998) and upregulate hypertrophic markers such as alkaline phosphatase and collagen type 10 in cultured chicken chondrocytes [65]. In addition to COL10A1, AA has been shown to promote COL1 synthesis in foetal bovine chondrocytes [46,60]. Promotion of COL1A1 expression has also been demonstrated in human chondrocytes [56], human fibroblasts [66,67,68], guinea pig articular cartilage explants [58], and ATDC5 cells [57]. The osteogenic effect associated with AA has also been demonstrated in stem cells from human deciduous teeth, where AA increased their chondrogenic and osteogenic potential and increased the secretion of the metabolites PTH, ALPL, and OPN, which are responsible for bone growth and development [18].

In our study, we first examined the expression of COL1A1, which is characteristic of pathological situations such as OA [46], and endochondral ossification [35]. The presence of AA in cell culture medium did not increase the expression of COL1A1 in cAMSCs, but the absence of AA downregulated the expression of COL1 after 7 days of culture, compared with the negative control. The downregulation of COL1A1 might be related to a lack of upregulation of RUNX2 (transcription factor of COL10A1). Langenbach et al. have shown that COL1A1 synthesis leads to the expression of RUNX2 via ERK1/2 and MAPK signalling pathways, thereby promoting osteogenic differentiation of stem cells in vitro [17]. This correlates with our observation in SF cells cultured for 7 days in the absence of AA, where downregulation of COL1A1 was not accompanied by upregulation of RUNX2. However, this observation should be further investigated. Analysis of SF cells revealed that the expression of RUNX2 was higher in SF-AA cells on day 7 than on day 3. Moreover, the expression of RUNX2 on day 7 was higher in SF-AA than in SF-NoAA cells, suggesting that the expression of RUNX2 in SF cells is affected by both AA and the time of cultivation. Compared with the negative control, MMP13 was upregulated in all groups of cAMSCs except SF-NoAA-3D, suggesting that AA promotes MMP13 expression in cAMSCs grown on SF films. This is supported by the results of other studies in other cell types, the ATDC5 cell line [57] and MC3T3-E1 osteoblast cells [69], which show a correlation between AA and the increase in mRNA expression of MMP13. Interestingly, the expression of MEF2C was similar in all groups of cells, indicating that neither SF nor AA affects its expression. Summarising the effect of AA on gene expression of chondrogenic genes only in SF cells AA appears to downregulate the chondrogenic genes SOX9, COMP, and ACAN, as shown by the downregulation of SOX9 and COMP on day 3 in SF-AA cells compared to SF-NoAA cells. In addition, downregulation of the ACAN gene was stronger between days 3 and 7 in SF-AA cells compared to SF-NoAA cells. In contrast to the chondrogenic genes, AA upregulated the hypertrophic gene RUNX2, as shown by the upregulation of RUNX2 in SF-AA cells between days 3 and 7 and also the upregulation of RUNX2 on day 7 in SF-AA cells compared to SF-NoAA cells. This suggests that when culturing cAMSCs on SF films in a growth medium, AA may not be advantageous if chondrogenic gene expression is desired and hypertrophic gene expression, namely, RUNX2, is to be avoided.

Overall, the results of this gene expression study showed that the combination of SF and the optional addition of AA to the cell culture medium appears to be important for modulation of expression of chondrogenic and hypertrophic genes. To our knowledge, this is the first study to investigate the combined effect of SF and AA on chondrogenic and hypertrophic gene expression of cAMSCs triggered by SF. This opens the possibility to further explore the options to induce the desired chondrogenic phenotype of MSCs, e.g., by using a hypoxic atmosphere, adding specific proteins or hormones to the culture medium, or growing the cells in a 3D SF environment, e.g., SF hydrogel. In future studies, protein analysis of the extracellular matrix and the relationships between gene expression and matrix deposition should also be explored.

## 5. Conclusions

In the present study, we investigated the effect of AA-enriched growth medium on the proliferation of cAMSCs grown on a plastic surface and on the differentiation of cAMSCs grown on SF. We have shown that AA significantly improves the proliferation potential of cAMSCs grown on plastic surfaces, without affecting cell viability, indicating that AA may be used for faster expansion of cAMSCs. Additionally, we have shown that AA modulates the expression profile of chondrogenic and hypertrophic genes when grown on SF, which provides the basis for future studies aimed at exploring the possibilities of further controlling the chondrogenic or hypertrophic phenotype of MSCs, with the goal of developing specifically modified cells with potential clinical relevance.

Modulation of proliferation and chondrogenic differentiation of canine MSCs could therefore help navigate the future preparation of MSCs for therapeutic use in veterinary medicine.

## Figures and Tables

**Table 1:** Data on antibodies and dilutions used for flow cytometry analysis in the study.

Surface Marker	Conjugation	Antibody Clone	Isotype	Target Species	Catalogue Number	Source	Antibody Dilution per 1 × 10^6^ Cells
CD44	APC	IM7	Rat IgG2b	Mouse, human	103012	Biolegend, USA	1:67
CD90	PE	YKIX337.217	Rat IgG2b	Dog	12-5900-42	eBioscience, USA	1:20
CD29	FITC	MEM-101A	Mouse IgG1	Dog, Human, Pig	MA1-19566	ThermoFisher Scientific, USA	1:5
CD34	FITC	581	Mouse IgG1	Human	60013FI	Stemcell technologies, Vancouver, BC, Canada	1:20

CD: cluster of differentiation, FITC: fluorescein isothiocyanate, APC: allophycoerythrin, PE: phycoerythrin.

**Table 2:** Names of the cell cultures, cell culture media, cell seeding surfaces and densities, passages, time of culturing, and culture conditions used in the study.

Cell Culture Name	Cell Seeding Surface	Culture Medium	Culture Time	Cell Seeding Density	Culture Conditions
SF-AA-3D	SF film (12.5 mg/mL)	Cell culture medium with ascorbic acid	3 days	10^4^ per cm^2^	37 °C, 5% CO_2_
SF-AA-7D	SF film (12.5 mg/mL)	Cell culture medium with ascorbic acid	7 days	10^4^ per cm^2^	37 °C, 5% CO_2_
SF-NoAA-3D	SF film (12.5 mg/mL)	Cell culture medium without ascorbic acid	3 days	10^4^ per cm^2^	37 °C, 5% CO_2_
SF-NoAA-7D	SF film (12.5 mg/mL)	Cell culture medium without ascorbic acid	7 days	10^4^ per cm^2^	37 °C, 5% CO_2_
POZ K	Standard polystyrene	Chondrogenic medium	P4	5 µL droplets of 4 × 10^4^ cells	37 °C, 5% CO_2_, high humidity
NEG K	Standard polystyrene	Cell culture medium	P4	10^4^ per cm^2^	37 °C, 5% CO_2_

SF: silk fibroin; AA: ascorbic acid; NoAA: no ascorbic acid; 7D: 7 days; 3D: 3 days.

**Table 3:** Gene symbols and names, assay identification numbers, and gene roles in chondrogenesis.

Gene Symbol	Gene Name	Assay ID	Gene Role in Chondrogenesis
COL2A1	Collagen type II, alpha 1	Cf02622868_m1	Cartilage-specific marker gene [30]
SOX9	SRY (sex determining region Y)-box9	cf02625134_g1	The first transcription factor, essential for chondrocyte differentiation and cartilage formation [30]
ACAN	Aggrecan	Cf02674826_m1	Cartilage-specific marker gene [31]
COMP	Cartilage oligomeric matrix protein	Cf02690298_g1	Cartilage-specific marker gene [32]
COL1A1	Collagen type I, alpha 1	Cf01076765_m1	A main component of fibrocartilage characteristic of OA [33,34] and distinctive of endochondral ossification [35]
MMP13	Matrix Metalloproteinase 13 (Collagenase 3)	Cf02741638_m1	Hypertrophic marker gene known to break down collagen type 2 [36,37].
RUNX2	Runt-related transcription factor 2	Cf02694692_m1	Direct transcriptional factor of COL10A1 during chondrogenesis [38] and hypertrophic marker gene [32,39,40]
MEF2C	Myocyte enhancer factor 2C	Cf02696950_m1	Hypertrophic marker gene [39,41]
TBP	TATA box binding protein	Cf02637231_m1	Reference gene

## Data Availability

The datasets used and/or analysed during the current study are available from the author on reasonable request.
